# Combined Mutational and Spectroscopic Study on the Calcium-Related Kinetic Effects on the VirChR1 Photocycle

**DOI:** 10.1021/acs.jpcb.4c08416

**Published:** 2025-03-10

**Authors:** Gerrit H. U. Lamm, Dmitrii Zabelskii, Taras Balandin, Valentin Gordeliy, Josef Wachtveitl

**Affiliations:** † Institute of Physical and Theoretical Chemistry, Goethe University Frankfurt, 60438 Frankfurt Am Main, Germany; ‡ European XFEL, 22869 Schenefeld, Germany; § Institute of Biological Information Processing (IBI-7: Structural Biochemistry), Forschungszentrum Jülich, 52428 Jülich, Germany; ∥ JuStruct: Jülich Center for Structural Biology, Forschungszentrum Jülich, 52428 Jülich, Germany; ⊥ Univ. Grenoble Alpes, CEA, CNRS, Institute de Biologie Structurale (IBS), 38000 Grenoble, France

## Abstract

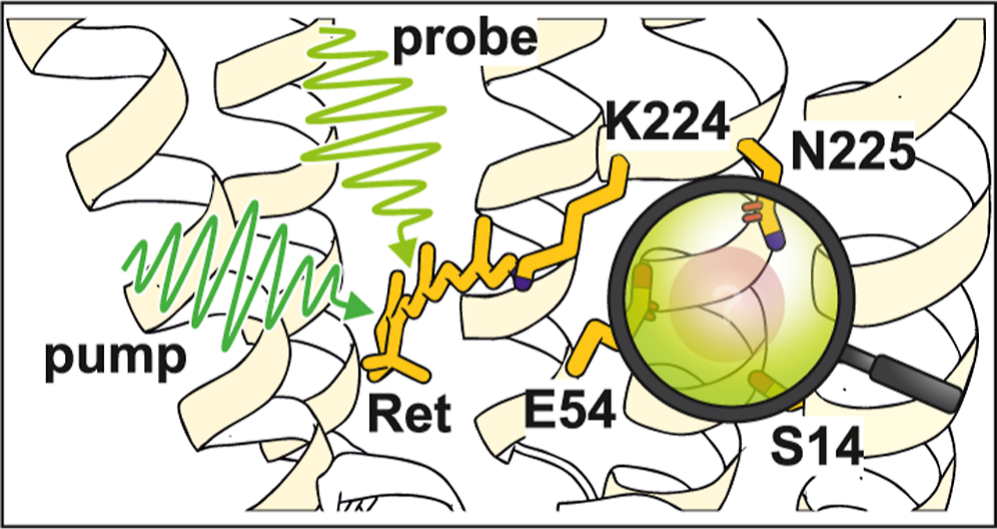

The viral rhodopsin 1 subfamily consists of microbial rhodopsins, such as VirChR1, with a light-gated cation channeling functionality, which is inhibited by calcium. For VirChR1, S14, E54, and N225 have been proposed as key residues for calcium binding. They form a highly conserved SEN-triad in channelrhodopsins near the functionally important central gate. Here, we present a time-resolved UV/vis spectroscopic study on the VirChR1 variants S14A, E54A, and N225A in a calcium-dependent manner. Comparison with the calcium-associated effects observed for the wild type shed light on the role of the respective residues for the calcium interaction. While S14A shows less pronounced, yet similar, signals, indicative of a reduced calcium affinity, E54A exhibits nearly calcium-independent photocycle kinetics, highlighting its crucial role for calcium binding. The N225A variant shows altered photocycle kinetics, in both the absence and presence of calcium, demonstrating its critical role in the formation of the functionally important central gate in VirChR1.

## Introduction

Microbial rhodopsins compose one of the largest families of light-sensitive proteins, the so-called photoreceptors, and the respective opsin genes are found among various domains of life, such as archaea, bacteria, fungi, or even viruses.^1−5^ Despite the wide variety of potential origins, the main structural features, like the seven transmembrane α-helices or the covalently attached retinal chromophore, are conserved among all microbial rhodopsins. In general, solar energy is absorbed to trigger biochemical processes important for cellular life.^6−8^ This includes sensory function as illustrated by, e.g., sensory rhodopsin I and II from *Halobacterium salinarum*,^9^ while ion-transporting (bacteriorhodopsin from *H. salinarum* (*Hs*BR)^10^ and many others^2,11,12^) or ion-translocating (channelrhodopsin-2 from *Chlamydomonas reinhardtii* (*Cr*ChR2)^13^ and anion channelrhodopsins^14,15^) functions are more prominently represented within the protein family, compared to, e.g., enzymerhodopsins.^16^ The development of optogenetics, in which microbial rhodopsins are used for light-triggered manipulation of membrane potentials in living organisms,^17−21^ as well as for ion conduction toward the cytoplasm and in organelles,^22^ further increased research efforts. Besides discovering novel applications, additional focus was put on gaining detailed knowledge about the molecular mechanism resulting in the respective protein functionality. Ultimately this knowledge led to non-native protein variants with adjusted properties and therefore better or wider applicability within optogenetics. In the case of *Cr*ChR2, protein engineering resulted, e.g., in accelerated photocurrent kinetics,^23^ or changed ion selectivity.^24^ Recently a subfamily of viral rhodopsins 1 (VR1) was discovered. Functional studies revealed ion channel functionality, while at least one representative (VirChR1) showed a strong calcium interaction in both electrophysiology and spectroscopy. It was shown that the ion channel functionality is abolished once a certain threshold calcium concentration is reached.^5,25^ Our recent initial spectroscopic characterization showed that VirChR1 still undergoes a photocycle when ion translocation activity is abolished by calcium. The photocycle of the blocked channel is significantly altered compared to that of the active channel. Accordingly, two different photocycle schemes were derived, where the presence of spectrally and kinetically different intermediates P_6_^Ca^ and P_7_ reflects the clear alterations.^26^ Especially a detailed understanding of the calcium interaction is of particular interest due to the high physiological relevance of calcium given by its important role in many central biochemical processes.^27−30^

**Figure 1:**
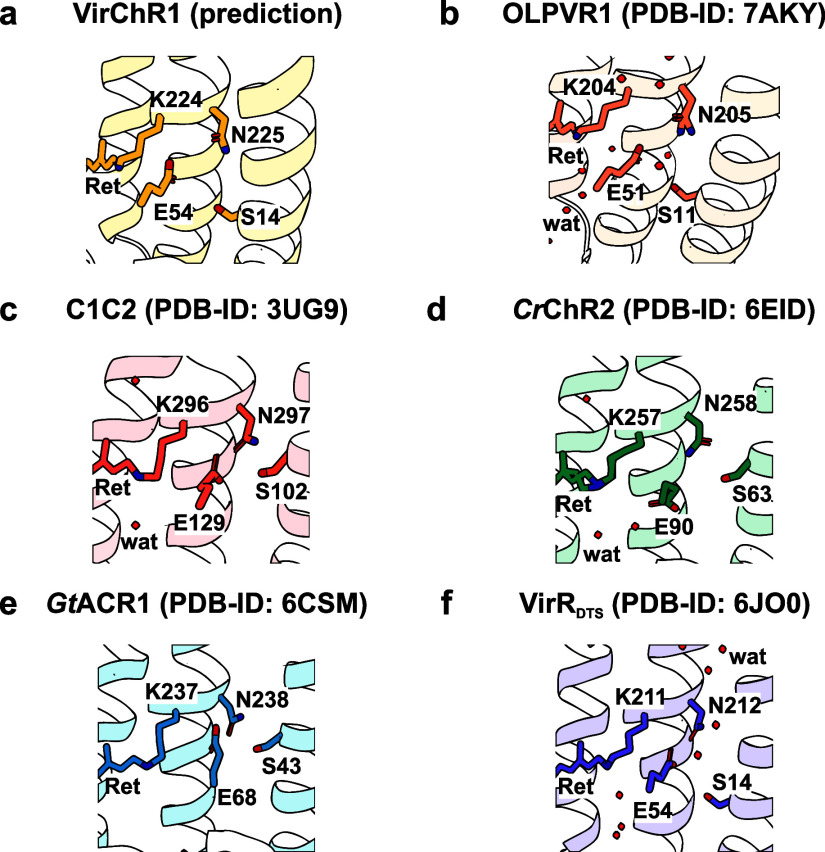
Structural representation of the SEN-triad, which is conserved among known channelrhodopsins. (a) Structure prediction obtained using AlphaFold2^31,32^ realized via ColabFold.^33^ All other shown structures represent experimentally solved X-ray structures of (b) OLPVR1,^5^ (c) C1C2 (a chimera of channelrhodopsins 1 and 2 from *Chlamydomonas reinhardtii*),^34^ (d) *Cr*ChR2,^35^ (e) *Gt*ACR1,^14^ and (f) VirR_DTS_.^4^ Structural data were assessed via the PDB IDs mentioned in the figure. Red dots indicate water molecules. All structural visualizations were created using PyMOL software.

Within their first study, Zabelskii et al.^5^ proposed a potential calcium-binding site within VR1 subfamily members, formed by residues S14, E54, and N225 (VirChR1 numbering, Figure 1). Besides representing a potential calcium-binding site in VirChR1, this SEN-triad is highly conserved among channelrhodopsins (Figure 1) with the glutamate and the asparagine residues forming the functionally important central gate (CG),^5^ similar to residues E90 and N258 in *Cr*ChR2. Variations within the CG have shown strong effects on channeling kinetics and ion selectivity for various known channelrhodopsins. Thus, the conserved SEN-triad is not only involved in the formation of the CG but also decisive for ion selectivity. Different SEN-triad variants of cation channelrhodopsins have been investigated in the past. Turning *Cr*ChR2 into a selective chloride channel (E90R),^24^ affecting its proton- and sodium-mediated photocurrents (E90A),^36^ or reduction of calcium selectivity for C1C2 (S102D and E129A),^24,34,37,38^ showed the link of the SEN-triad to ion selectivity. Apparently, this is only the case for cation channelrhodopsins, as indicated by variants of the anion channelrhodopsin from *Guillardia theta* (*Gt*ACR1) (E68Q and N239Q), as they remained selective for anions.^5,14^ This fits well with the SEN-triad of VirChR1, which has been proposed as the calcium-binding site, making its impact an important aspect to investigate. Accordingly, upon removal of charged residues/carboxylates, a modulation of the calcium-related kinetic effects toward becoming calcium-insensitive is expected. In order to gain further insights into this calcium interaction, we investigated alanine variants of all residues of the proposed calcium-binding triad in VirChR1 (S14A, E54A, and N225A) by time-resolved UV/vis spectroscopy on the late nanosecond to second time scale. Based on a comparison of the experimental data for all three investigated variants obtained within this study and our earlier study on the wt,^26^ conclusions of the overall involvement in the calcium interaction were drawn.

## Methods

### Protein Expression

The *E. coli* codon-optimized VirChR1 gene was synthesized commercially (Eurofins). The nucleotide sequence was optimized for *E. coli* expression using the GeneOptimizer (Life Technologies). The gene, together with the 5′ ribosome-binding sites and the 3′ extension coding additional LEHHHHHH* tag (His-tag), was introduced into the pEKT expression vector (Novagen) via Ndel and Xhol restriction sites and verified by sequencing. The protein was additionally supplemented with BRIL protein on the N-terminus of the protein to improve the protein folding and expression level.^39^ The S14A, E54A, and N225A variants were prepared by site-directed mutagenization and verified by sequencing (Eurofins Genomics). The proteins were expressed as described previously.^5,26^*E. coli* cells of strain C41 (StabyCodon T7, Eurogentech) were transformed with the expression plasmid. Transformed cells were grown in shaking baffled flasks in an autoinducing medium ZYP-5052 containing 100 mg/L kanamycin at 37 °C. When the OD_600_ in the growing bacterial culture was 0.8–1.0 (glucose level <10 mg/mL), 10 μM all-trans retinal (Sigma-Aldrich) and 1 mM isopropyl-β-*d*-1-thiogalactopyranoside were added, the incubation temperature was reduced to 20 °C, and it was incubated for 18 h. After incubation, cells were collected by centrifugation (5000*g*, 30 min) and disrupted in an M-110P lab homogenizer (Microfluidics) at 20,000 p.s.i. in a buffer containing 20 mM Tris–HCl, pH 8.0, with 50 mg/mL DNase I (Sigma-Aldrich). The membrane fraction of the cell lysate was isolated by ultracentrifugation at 90,000*g* for 1 h at 4 °C (Type 70 Ti fixed-angle titanium rotor, Beckmann). The pellet was resuspended in a buffer containing 20 mM NaH_2_PO_4_/Na_2_HPO_4_, pH 8.0, 0.1 M NaCl, and 1% *n*-dodecyl-β-d-maltoside (DDM, Anatrace, Affymetrix) and stirred for 18 h for solubilization. The insoluble fraction was removed by ultracentrifugation at 90,000*g* for 1 h at 4 °C. The supernatant was loaded on a Ni-NTA column (Qiagen) and washed with a buffer containing 20 mM Tris, pH 8.0, 200 mM NaCl, 20 mM imidazole, and 0.05% DDM. The eluate was subjected to size-exclusion chromatography on a 20 mL Superdex 200i 10/300 GL column (GE Healthcare Life Sciences) in a buffer containing 20 mM Tris, pH 8.0, 150 mM NaCl, and 0.05% DDM. Protein-containing fractions were pooled and concentrated up to 10 mg/mL. The BRIL fusion protein and the His-tag were removed using a thrombin endonuclease using the following protocol. The concentrated protein was mixed with 0.1 U/mL thrombin (Merck) in a 10× thrombin cleavage buffer of 200 mM Tris, 1.5 M NaCl, and 25 mM CaCl_2_, pH 8.4. The mixing ratio was 1 μL of 0.1 U/mL thrombin for 1 mg of VirChR1-BRIL protein. 0.01% DDM was added to the reaction, and the buffer was diluted 10-fold. The reaction mixture was left for 72 h in the dark at 20 °C to ensure complete cleavage. The reaction mixture was loaded onto the Ni-NTA column (Qiagen) to remove noncleaved protein and washed with 20 mM Tris, pH 8.0, 200 mM NaCl, and 0.05% DDM. The eluate was subjected to the second round of size-exclusion chromatography on a 20 mL Superdex 200i 10/300 GL column in 20 mM Tris, pH 8.0, 200 mM NaCl, and 0.05% DDM buffer. The purified VirChR1 was pooled and concentrated to 40 mg/mL to serve as the stock solution for the spectroscopic experiments.

### Sample Preparation

For transient flash photolysis experiments, tiny amounts of the stock solution of the respective protein were diluted in a buffer containing 20 mM Tris, 150 mM NaCl, and 0.05% DDM. The pH of the buffer was set to 8.0. Protein concentration was adjusted to be equivalent to an optical density of ≈1 at an optical path length of 10 mm. Prior to and after each measurement, an absorption spectrum was measured to check sample quality using an absorption spectrometer (Specord 600, Analytik Jena).

### Prediction of the VirChR1 wt Structure

The structure of VirChR1 was modeled in two steps. First, the structure of the opsin dimer has been predicted using AlphaFold2^31,32^ in ColabFold.^33^ A detailed overview of the used parameters is given in Table S1.

The three resulting models have shown similar prediction scores, as evidenced in predicted local distance difference test (pLDDT) scores (Figure S1). For the second step, we have selected the model with the highest ranking (pLDDT = 87.25). The retinal cofactor was added to the predicted structure using AlphaFill.^40^ The resulting PDB file was used for visualization only.

### Transient UV/Vis Flash Photolysis Spectroscopy

A home-built transient flash photolysis setup was used to measure the photocycle kinetics from the late hundreds of nanoseconds up to several seconds. Within this setup, a nanosecond Nd/YAG laser (SpitLight 600, InnoLas Laser) pumped an optical parametric oscillator (preciScan, GWU-Lasertechnik). The output wavelength of the OPO was set to 510 nm for all measurements, and the output energy was adjusted to ≈2.2 mJ/cm^2^. Furthermore, the setup consists of two identical monochromators (500 nm blaze, 1200 L/mm), one placed in front and one after the sample. The white light of a xenon or mercury–xenon lamp (LC-08, Hamamatsu) was used to monitor the laser flash-induced absorption changes. For signal detection, a PMT (Photosensor H6780-02, Hamamatsu) was mounted directly onto the second monochromator. Afterward, the measured absorption changes were digitized and recorded using two identical oscilloscopes (PicoScope 5244B/D, Pico Technology) with overlapping time scales. For each probing wavelength, 30 acquisitions were measured to increase the S/N ratio. The obtained large raw data files were reduced using moving averaging^41^ with a combined linear and logarithmic time scale prior to further analysis. The reduced time scale was set to be linearly spaced until 5 μs with a spacing of 56 ns. After 5 μs, the time scale was exponentially spaced, whereby the number of exponentially spaced steps considered varies depending on the photocycle duration. Due to an artifact stemming from the coupling of the two oscilloscopes, the following time points have been excluded from the respective measurement (Table 1).

**Table 1:** Time Points Excluded from the Measurements due to an Artifact of the Coupling of Two Oscilloscopes

sample	0 mM	1 mM	4 mM	60 mM
S14A	464.5 ms	461.5 ms	461.5 ms	461.5 ms
	468.2 ms	468.2 ms	468.2 ms	468.2 ms
E54A	459.3 ms	459.3 ms	459.3 ms	459.3 ms
	467.2 ms	467.2 ms	467.2 ms	467.2 ms
N225A	462.5 ms	462.5 ms	462.5 ms	461.9 ms
	466.9 ms	466.9 ms	466.9 ms	466.9 ms

The samples were measured in a 2 × 10 mm quartz cuvette. The pump and probe beam were aligned perpendicular to each other and hit the sample in a 90° geometry. The 2 mm optical path through the cuvette was used for sample excitation, and the 10 mm optical path through the cuvette was used to probe induced absorption changes. For CaCl_2_ concentrations of 0 and 60 mM, absorption changes were monitored in the spectral range from 330 to 700 nm with 10 nm steps. For intermediate concentrations of 1 and 4 mM CaCl_2_, absorption changes were probed at wavelengths characteristic for the observed photocycle intermediates (340, 380, 410, 480, 500, 560, 570, and 620 nm).

### Kinetic Analysis of Time-Resolved Spectroscopic Data

Time-resolved spectroscopic data was analyzed using OPTIMUS software,^42^ freely available at www.optimusfit.org. Data sets in the presence of 0 and 60 mM CaCl_2_ were subjected to the model-free lifetime distribution analysis (LDA) approach. As a result, the lifetime distributions of the individual photointermediate transitions were obtained, summarized in the corresponding lifetime distribution map (LDM). Lifetimes mentioned throughout the article have been derived from the maximum of the respective lifetime distribution and are therefore given as approximate values. For further comparability, and especially for the data obtained in the presence of 1 and 4 mM CaCl_2_, global lifetime analysis (GLA) was performed, again using OPTIMUS software. The via GLA-determined lifetimes are indicated in the respective LDM via dashed lines.

LDM calculations were performed with the following parameters within OPTIMUS software:^42^ In the case of VirChR1 N225A under permeable channel conditions, the LDM calculation with the parameters mentioned above did not provide a proper fit of the experimental data on the later time scales. This is due to the duration of the measurement and signals being constant in amplitude over several orders of magnitude in time. Accordingly, an additional LDM was calculated with the parameters adjusted as given in the *N225A* permeable channel column of Table 2. The results of the kinetic analysis of the obtained time-resolved spectroscopic data are provided in the Supporting Information.

**Table 2:** Parameters Used for LDA in OPTIMUS

parameter	standard setting	N225A permeable channel
regularization factors	50	50
number of lifetimes	500	500
start lifetime	0.0007 ms	0.1 ms
end lifetime	3× last exp. time point	1,000,000 ms

## Results and Discussion

**Figure 2:**
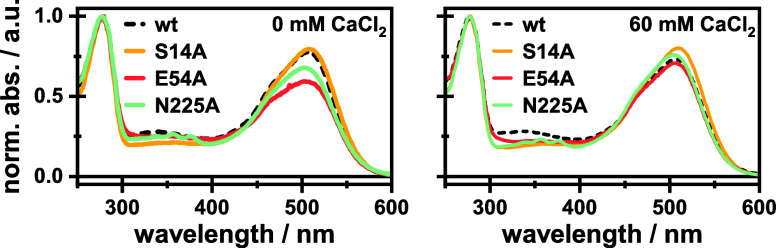
Absorption spectra of wt and investigated variants in the absence (left) and presence (right) of CaCl_2_.

To gain further insight into the calcium sensitivity of the VirChR1 photocycle kinetics, all members of the proposed calcium-binding site (S14-E54-N225,^5^Figure 1a) were each individually mutated to alanine. The absorption spectra of the respective variants are shown in Figure 2. None of the introduced mutations led to pronounced spectral shifts in the absorption spectrum. Accordingly, we assume that the retinal chromophore and its direct environment remain unaffected. Based on the calcium dependence of VirChR1 cation channeling functionality reported by Zabelskii et al.,^5^ we define two cases to be considered: (i) permeable channel conditions (0 mM CaCl_2_): cation translocation by VirChR1 upon excitation and (ii) blocked channel conditions (60 mM CaCl_2_): cation translocation by VirChR1 is abolished. We first check for the putative impact of the mutations on VirChR1 under permeable channel conditions. Following on that, a direct comparison of the obtained data and its corresponding lifetime distribution maps (LDMs) of the wt and the different variants under blocked channel conditions (shown in the Supporting Information) is performed. This allows us to draw conclusions about the role of each respective residue in the observed calcium-dependent kinetic effects on the VirChR1 photocycle.

### Kinetic Effects under Permeable Channel Conditions

**Figure 3:**
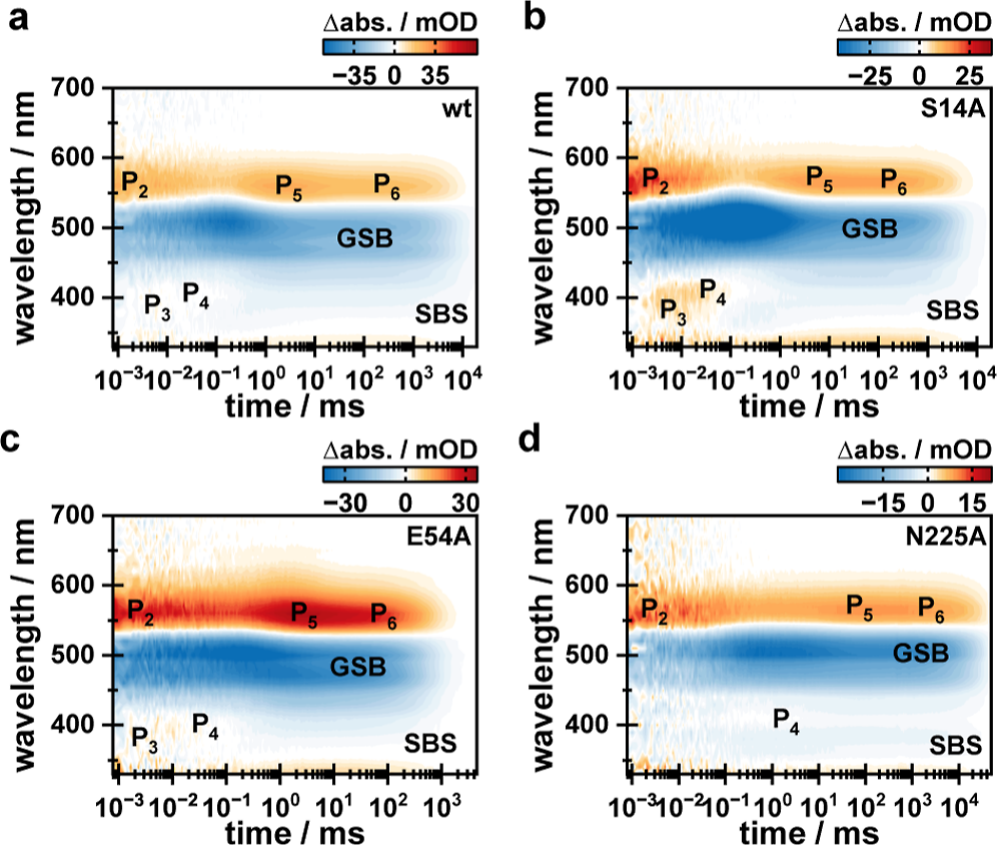
Flash photolysis data of (a) wt and (b) variants S14A, (c) E54A, and (d) N225A obtained under open channel conditions (pH 8.0, 150 mM NaCl, 0 mM CaCl_2_). wt data were taken from Lamm et al., *J. Phys. Chem. Lett.*, **2024**, *15*, 5510–5516.^26^

**Figure 4:**
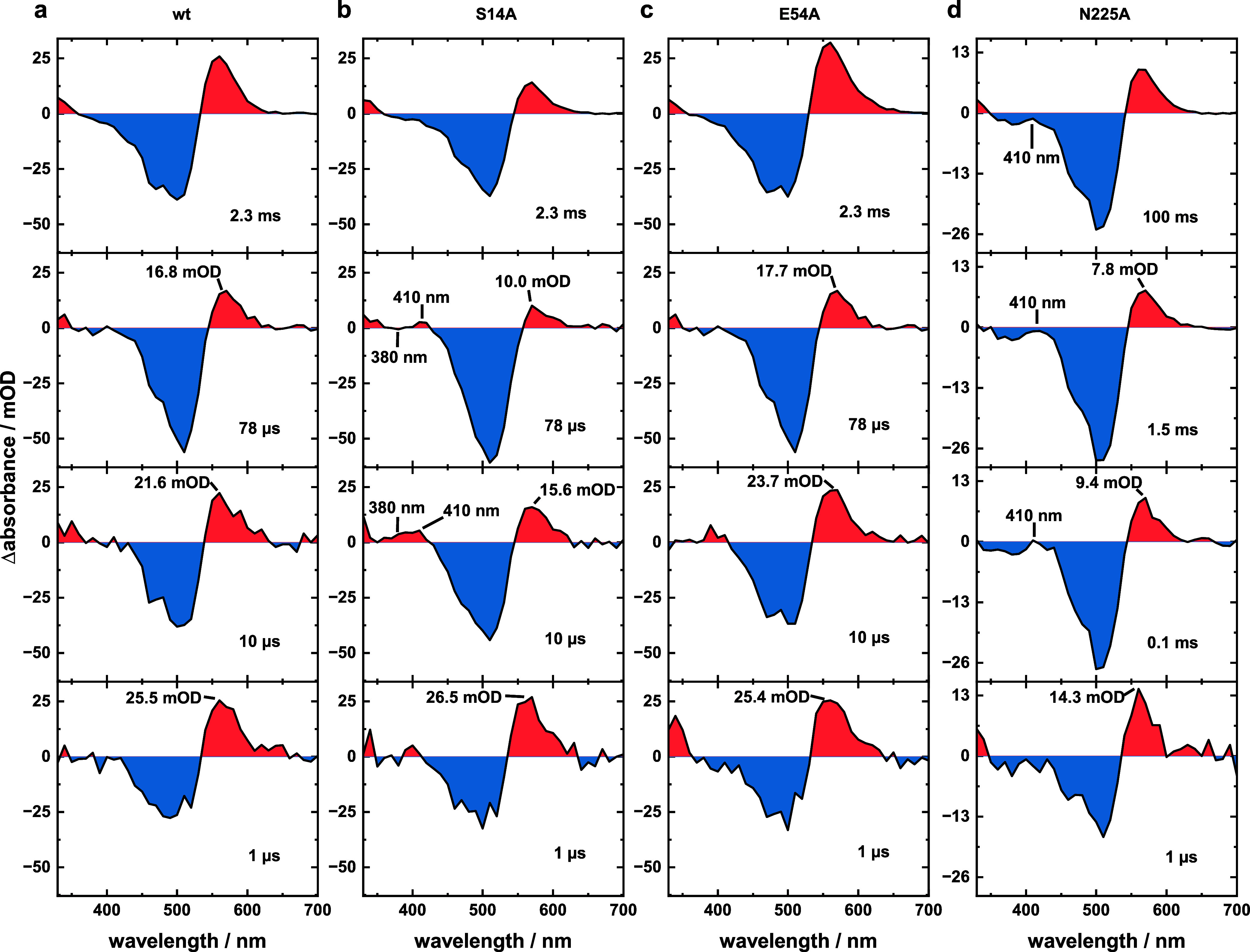
Difference absorption spectra at specific time points for (a) wt and variants (b) S14A, (c) E54A, and (d) N225A obtained under permeable channel conditions. wt data are taken from Lamm et al., *J. Phys. Chem. Lett.*, **2024**, *15*, 5510–5516.^26^

To facilitate the comparison, the wt data are reprinted in Figures 3a and 4a, and the photocycle kinetics is briefly introduced. For a more detailed discussion, we refer to the original publication.^26^ On this time scale, the dynamics starts with the red-shifted P_2_ intermediate, followed by the blue-shifted intermediates P_3_ and P_4_. Given the weak amplitude of P_3_ and P_4_ and the persistent presence of P_2_, an equilibrium between P_2_ and either P_3_ or P_4_ was assumed. Subsequently, another red-shifted intermediate (P_5_) was observed. This was followed by the P_6_ intermediate, which showed only minor but distinguishable spectral differences from that of P_5_. At later times, the photocycle is concluded, indicated by the decay of the P_6_ intermediate, the ground-state bleach (GSB), and the second-bright-state signature (SBS), which indicates a 13-cis retinal configuration throughout the photocycle.^26,43,44^ Based on the observed calcium-related kinetic effects and the determined τ_off_ value (∼150 ms),^5^ it was assumed that intermediates P_5_ and P_6_ are associated with the ion channeling functionality.

As evident by the comparison of the data sets shown in Figure 3, all three variants mostly reassemble the photocycle scheme of the wt. Nevertheless, certain differences in photocycle duration and the behavior of the first photocycle intermediates P_2_–P_4_ are evident. Both variants S14A and E54A cause a significant acceleration of the photocycle kinetics by factors of 2 and 12 in comparison to the wt, while the photocycle duration is elongated by a factor of 3 in the case of the N225A variant. The lifetimes determined for all observed intermediate state transitions show that the change in photocycle duration for variants S14A and E54A is only reflected in the last lifetime describing the recovery of the dark state, while variant N225A generally causes stronger changes to the photocycle scheme (Table S2). Furthermore, the equilibria between intermediate P_2_ and either P_3_ or P_4_ are affected by the introduced mutations. Variants S14A and E54A cause changes in the amplitude ratio of the mentioned intermediates. This is best illustrated when comparing the P_2_ intensity for the wt, S14A, and E54A at time points 1 and 78 μs after excitation. With regard to the intensity at 1 μs, ∼ 66% remain for the wt, ∼38% for S14A, and ∼70% for E54A (Figure 4a–c).

In agreement with that, S14A shows the strongest signals of both P_3_ and P_4_, and a comparison of the difference absorption spectra at 10 and 78 μs clearly resolves that there are two different blue-shifted intermediates populated during the VirChR1 photocycle (Figure 4b). The faster decaying one (P_3_) is located at ∼380 nm, while the subsequent P_4_ intermediate is located at ∼410 nm. Those intermediates are hard to distinguish for all other samples since they mainly cause a compensation of ground-state bleach (GSB) intensity. Yet, the pattern describing the behavior of intermediates P_2_ to P_4_ is observed in the LDMs of the wt, S14A, and E54A variants under all considered conditions (Figure S9, dashed boxes). The kinetic behavior as well as the amount of observed blue-shifted intermediates differs for the N225A variant. A single, weak blue-shifted intermediate was observed at ∼410 nm (Figure 4d), whose formation is decelerated, the population is elongated, and it conclusively decays significantly later at ∼15 ms. According to its spectral position, this is considered a P_4_ intermediate. Since P_3_ is obsolete in this case, processes occurring within the protein that cause the population of P_3_ likely involve residue N225, and thus, they become obsolete in the N225A variant. All of the points mentioned above illustrate that the SEN-triad affects the retinal *Schiff* base (RSB) deprotonation. In the case of variants S14A and E54A, this is done via a modulation of the mentioned equilibria, which were also observed for C1C2 in the past.^26,45^ For the E54A variant, the largest amount of P_2_ intensity remained at 78 μs after excitation, showing that the equilibria are shifted toward P_2_, thus implying that RSB deprotonation is not favored. The opposite is the case for the S14A variant, where the smallest remaining P_2_ intensity was observed at 78 μs after excitation. Accordingly, the equilibria are this time shifted toward the blue-shifted intermediates, and RSB deprotonation is more likely. With ∼54% remaining P_2_ intensity after 1.5 ms, N225A likely also possesses an equilibrium between intermediates P_2_ and P_4_, which is a bit further shifted toward P_4_ compared to the wt.

Several conclusions on this can be drawn based on a comparison with observations for other channelrhodopsins. The location within the protein and comparison with homologous residues in both *Cr*ChR2 and C1C2 rule out that the SEN-triad is directly involved in RSB deprotonation.^46,47^ More interesting is that a recent FTIR study on the other characterized VR1 representative OLPVR1 revealed similarities in the hydrogen bonding network between OLPVR1 and *Hs*BR.^48^ For instance, for *Hs*Br, it is known that changes to the electrostatic environment cause shifts of p*K*_a_ values relevant for RSB deprotonation.^49^ Accordingly, an impact of the SEN-triad on p*K*_a_ values can be expected, which would explain the observed changes with regard to the blue-shifted intermediates. One would expect that the E54A variant causes the largest effects due to the removal of its carboxylic side chain. It is known from various other rhodopsins that the respective analogues undergo a crucial flipping motion during the photocycle, causing large changes to the present hydrogen bonding networks and ultimately the opening of the channeling pore.^36,38,50,51^ This is further supported by a recent molecular dynamics (MD) study, which revealed changes of crucial p*K*_a_ values upon changes to the SEN-triad in C1C2.^52^ A special role for residue S14 might be possible since its hydroxyl group is oriented toward a pentagonal hydrogen bond cluster including both putative counterions D80 and D220. Yet, the hydroxyl group of residue S14 is located 5.3 Å apart from the nearest member of the cluster (D220) in the crystal structure of OLPVR1 (PDB-ID: 7AKY). The special role of N225A within all investigated variants is explained by its crucial role in forming the functionally important central gate (CG) with E54.^5^ From C1C2, we know that the serine and the glutamate compete for forming the CG with the asparagine.^50^ Thus, both variants S14A and E54A most likely possess an intact CG, which explains their overall wt-like photocycle kinetics. Replacement of the asparagine causes a disruption of the CG, as observed for the N297 V variant in C1C2, and the formation of an alternative CG involving residues E129 and E162.^52^ A similar behavior upon replacement of residue N225 is expected for VirChR1 as well and would explain the more significant changes to the photocycle kinetics compared to those of variants S14A and E54A.

### Kinetic Effects under Blocked Channel Conditions

Until now, the investigation of SEN-triad variants shows their involvement in setting the p*K*_a_ values that ultimately determine the efficiency of RSB deprotonation. Yet, the main target, which is their putative involvement in the characteristic calcium dependence of VirChR1, is not resolved. From both our previous study on the calcium dependence of the VirChR1 photocycle kinetics and the reported calcium dependence of VirChR1 functionality, it is known that the presence of >20 mM CaCl_2_ abolishes cation channeling functionality and significant changes to photocycle kinetics.^5,26^ This involves the formation of additional intermediates P_6_^Ca^ and P_7_ that are not observed in the absence of calcium. Thus, this can be seen as a spectroscopic readout for the characteristic calcium interaction.

To evaluate the role of the respective residue on the calcium interaction of VirChR1, the photocycle kinetics were measured in a calcium-dependent manner. For better comparability with our previous study on the wt, the same conditions were chosen for the variants as well. Since the calcium concentrations of 1 and 4 mM represented intermediate conditions, resulting in mixed kinetics for the wt, a focus was put on the measurements in the presence of 60 mM CaCl_2_, already introduced as blocked channel conditions.

**Figure 5:**
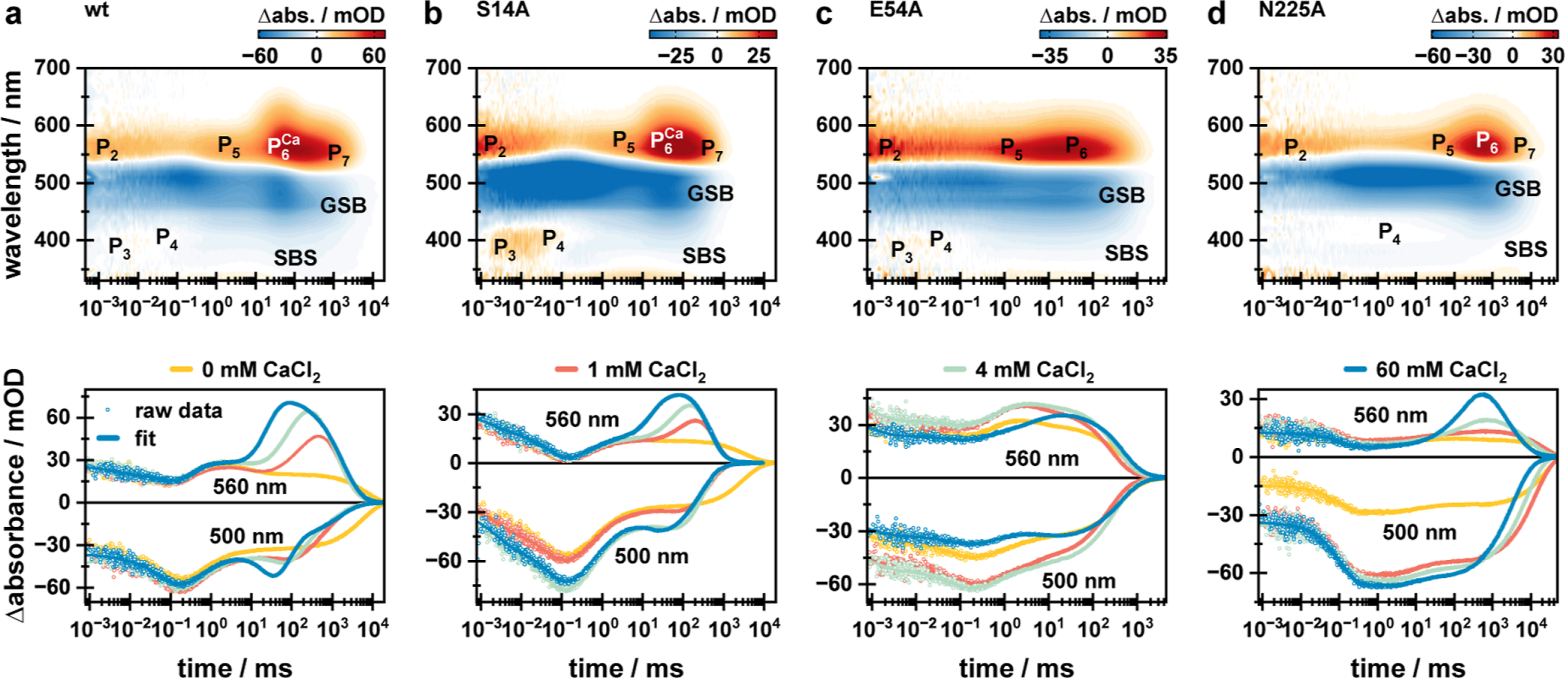
Flash photolysis data of the (a) wt and variants (b) S14A, (c) E54A, and (d) N225A obtained under blocked channel conditions. Upper panels show the whole data sets as 2D contour plots, while lower panels show transients at 500 and 560 nm, illustrating the strong calcium-dependent effect on both the GSB and intermediates P_6_^Ca^ and P_7_. wt data are taken from Lamm et al., *J. Phys. Chem. Lett.*, **2024**, *15*, 5510–5516.^26^

**Figure 6:**
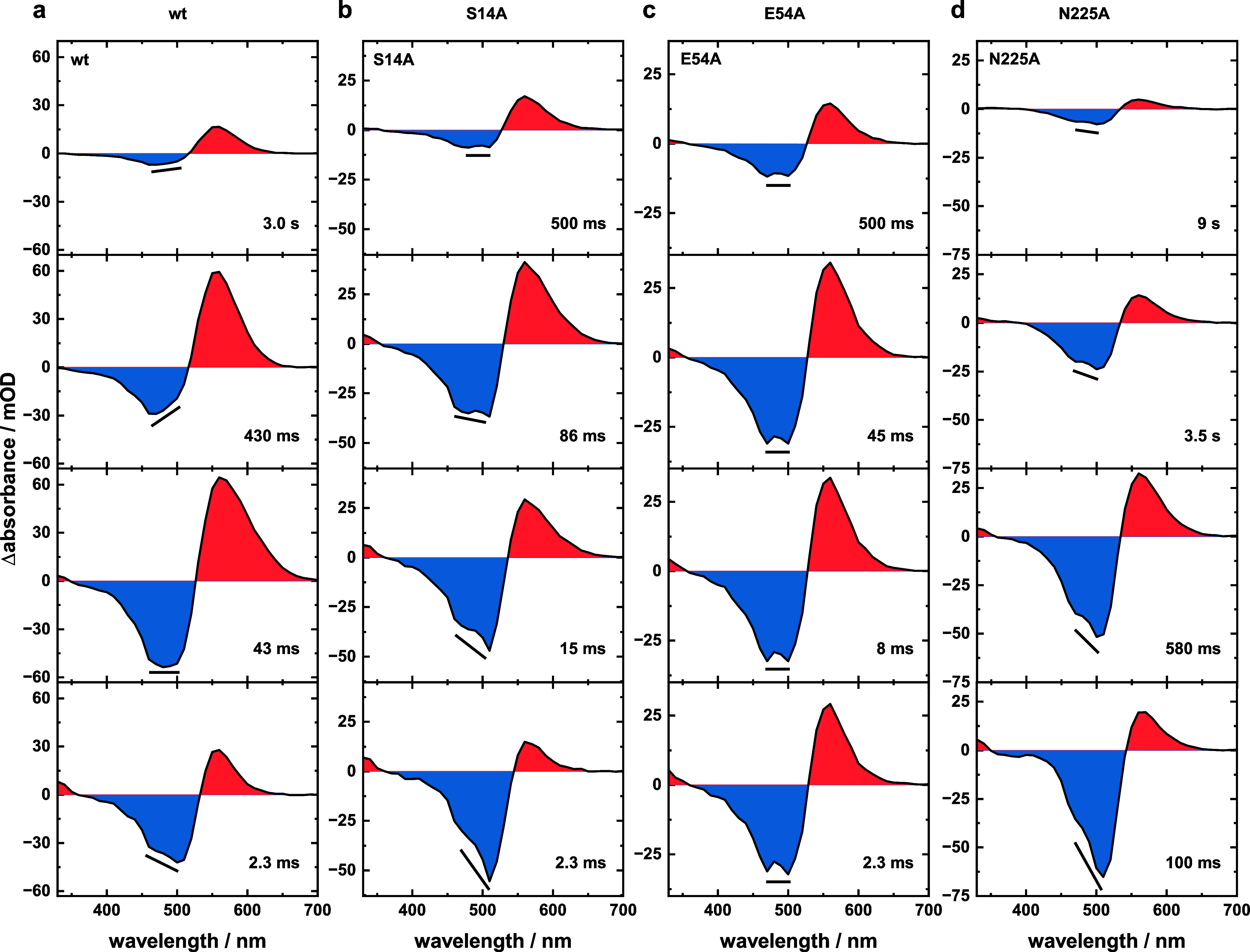
Difference absorption spectra at specific time points for (a) wt and variants (b) S14A, (c) E54A, and (d) N225A obtained under blocked channel conditions. Black lines illustrate the ratio of the two GSB maxima at 470 and 510 nm. wt data are reprinted in part from Lamm et al., *J. Phys. Chem. Lett.*, **2024**, *15*, 5510–5516.^26^

Also, as for permeable channel conditions, the effects observed for the wt are first briefly introduced. Addition of calcium to the buffer led to changes in the late photocycle intermediates (intermediates P_5_ to P_7_ in Figures 5a and 6a). The P_5_ intermediate is no longer followed by the P_6_ intermediate. Instead, a spectrally different P_6_^Ca^ was observed, succeeded by the last photocycle intermediate P_7_. In agreement with our previous study, the investigated variants showed calcium-related effects only for the late photocycle intermediates. Therefore, all trends already discussed for the equilibria , and  remained preserved compared to the same sample investigated under permeable channel conditions (lifetimes τ_1_–τ_3_ in Tables S2 and S3). Yet, a comparison of the obtained photocycle kinetics under permeable and blocked channel kinetics shows that all investigated variants possess altered photocycle kinetics upon the addition of calcium (Figures 3 and 5). Nevertheless, in each considered case, the extent is smaller compared to the wt.

Under blocked channel conditions, the S14A variant shows similar, but less pronounced, calcium-related effects as the wt (Figure 5b). Formation of P_6_^Ca^ and P_7_ was observed together with the modulation of the GSB intensity due to variation in the spectral position (Figures 5b and 6b). This is also reflected in the corresponding LDM (Figure S3). Direct comparison of the observed effects with the wt showed that the S14A variant under blocked channel conditions resembles the wt under intermediate conditions (1 and 4 mM CaCl_2_). For instance, spectral separation of P_6_^Ca^ is indicated both in the data and the LDM (Figure S3) and is similar to the wt at 1 mM CaCl_2_ yet not as pronounced as for the wt at 4 mM CaCl_2_.^26^ Accordingly, it was concluded that residue S14 takes part in the observed calcium-related effects while playing only a minor role (compare Figure 6a,b). This is also reflected by a decrease in calcium affinity. Taking into account that the extent of the effects is between those of the wt at 1 and 4 mM CaCl_2_, where the affinity value was determined to be ≈2.2 mM CaCl_2_,^5^ we expect a calcium affinity value somewhere in the range of 60 mM CaCl_2_ for the S14A variant. Of course, this needs to be verified in further studies in the future. The E54A variant is identified as a key player in VirChR1 calcium dependence due to almost calcium-independent photocycle kinetics under all investigated conditions (Figures 5c and 4c). The only observed effect is a shift in the amplitude ratio of intermediates P_5_ and P_6_. In the absence of calcium, this is dominated by P_5_, while P_6_ becomes stronger at 4 mM CaCl_2_, before being the dominant intermediate under blocked channel conditions (Figure 5c). This is associated with less modulation of the GSB intensity, as indicated by the 500 nm transient, which becomes more negative again in the range of 10–100 ms. This is temporally in line with the population of the P_6_ intermediate. Thus, P_6_ is red-shifted compared to P_5_. This reflects a behavior similar to that observed for the P_6_^Ca^ intermediate of the wt and the S14A variant. Additionally, the 560 nm transient showed small contributions indicative of a potential P_7_ intermediate. Yet, the observed effects are the weakest among all investigated samples, reflecting the E54A becoming almost calcium-independent. According to our data, we suggest a calcium affinity value higher than the 60 mM CaCl_2_ we propose for S14A, resulting in a calcium-independent version of VirChR1 under a majority of relevant conditions. N225A also showed calcium-related kinetic effects. In the first place, the observed gain in P_6_ intensity indicates similar calcium-dependent effects as for the other investigated samples (Figure 5d top). A more detailed look at the induced effects on the 500 nm transient (Figure 5d bottom) shows that the calcium-induced effects differ. Even in this case, the final red-shifted intermediate is affected further, providing evidence for its relation to the channeling function. According to the strong effects on the spectroscopic characteristics of the retinal chromophore, the calcium ion should be in close proximity to the retinal, thus reflecting a state with an open channel. Under blocked channel conditions, the amplitude of the P_6_ intermediate increases significantly (Figure 5). Yet, the modulation of the GSB intensity during the population of P_6_ differs from the trends observed for all other samples since the GSB does not increase in negative amplitude (Figure 5). Therefore, this P_6_ intermediate still overlaps with the GSB at ≈500 nm. Accordingly, this intermediate clearly expands into the red while maintaining its spectral contribution at ≈500 nm. Thus, this signal describes an intermediate, different from the P_6_^Ca^ intermediate observed for the wt and the S14A variant, showing a clearly altered calcium-related effect. Subsequently, a P_7_ intermediate is formed. Due to the altered spectral position of P_6_ in N225A, compared to P_6_^Ca^, the spectral indication for P_7_ is less clear in this case. The transition is indicated by the inhomogeneity of the positive lifetime distribution in the range of 540 to 650 nm (Figure S6). Accordingly, the red part of the signal decays earlier compared to the blue part of the signal. In addition, this transition (τ ≈ 1.4 s) is accompanied by a spectral broadening of the second-bright-state (SBS) signature.

As first discussed for the glutamate of the CG and later on expanded to the whole SEN-triad, this SEN-triad is highly conserved among channelrhodopsins and is always located at functionally extremely relevant positions (Figure 1).^5,53^ Alterations of the SEN-triad have been shown to cause significant changes in ion selectivity, spanning from an increase or decrease of selectivity up to changing the selectivity toward the anion chloride.^24,34,36−38,54^ A modulation of the calcium dependence of VirChR1 SEN-triad members is well reflected by our data (Figures 5 and 6). Nevertheless, a detailed comparison with the prototypic cation channelrhodopsins *Cr*ChR2 and C1C2 provides additional insights into the observed effects, especially for residues E54 and N225. Their role in forming the functionally important CG and the effects caused by the removal of each residue have already been discussed in the “Kinetic Effects under Permeable Channel Conditions” section. Of particular interest with regard to our findings is a recent MD study on sodium and calcium translocation by C1C2, revealing the importance of the electrostatic environment around the CG on cation translocation.^55^ This study has shown that cations are translocated via passing between adjunctant carboxylate residues on helices II and VII, while CG represents an energetic barrier. This is indicative of strong Ca^2+^ binding near the CG, leading to slowed down ion translocation or even to a partially blocked pore. This matches well with our observations for the E54A variant. It represents the glutamate of the CG and removal of the carboxylate, which showed by far the biggest impact on the calcium-dependent effects. Accordingly, the impact of the other two investigated variants on the calcium-dependent kinetic effects is smaller since no carboxylate side chain was introduced or removed. Furthermore, this study revealed a weak water-mediated interaction of residue S102 with translocated ions. We expect a similar weak involvement of residue S14 in the interaction with translocated ions since the S14A variant shows the smallest effects on the calcium dependence (Figure 5b).^55^ Residue S14 participates in the calcium effects but only weakly, as expected for a water-mediated interaction.

The findings presented in this study are in line with the influence of variants S102D and N297D on the ion selectivity of C1C2. The S102D variant slightly reduced the calcium selectivity, while all other cation-mediated currents remained unaffected.^34^ Despite the fact that direct statements on protein functionality have to be drawn carefully from the presented UV/vis spectroscopic data, we observed, in a similar fashion, a slight modulation of the calcium-related kinetic effects. Based on that and on the structural similarity of VirChR1 and C1C2, we propose that residue S102 plays the same role in both proteins and likely interacts with the cation via water-mediated hydrogen bonds. The direct C1C2 analogue of VirChR1 E54A is E129A, which also shows a strong effect on calcium selectivity while not altering proton or sodium selectivity.^34^ In agreement with the information gained for C1C2, the carboxylate of residue E54 plays a major role in the calcium interaction, while the flipped conformation of E54 is needed for pore opening and cation interaction.^36,38,50−52^ Yet, the E54A variant likely stays functional, similar to both *Cr*ChR2 E90A^36^ and C1C2 E129A.^34^ The glutamate and the serine compete for hydrogen bond formation with the arginine of the CG.^50^ Thus, they might be exchangeable for forming the CG, with the glutamate being the preferred interaction partner. At the moment, another calcium-binding rhodopsin called TAT rhodopsin and its calcium-binding ability are investigated in detail.^56^ While our previous study showed^26^ that the comparability of the calcium binding in VirChR1 and TAT rhodopsin is limited, it is worth mentioning that Sugimoto et al.^57^ identified the main residues for calcium binding in TAT rhodopsin (E54 and D227). Their mutational analysis revealed that removing one of the two coordinating residues significantly reduced calcium-related effects on the absorption spectrum but never resulted in a completely abolished calcium-binding ability,^57^ similar to our observations for the E54A variant. The sequence of carboxylates coordinating the cations is interrupted in the E54A variant; thus, the calcium affinity is significantly reduced. The introduction of an additional carboxylate by the N297D variant of C1C2 was shown to cause a gain in calcium affinity, whereby the removal of functional side chains (N297V) decreased the sodium affinity and led to a preference for protons.^55^ The CG was identified to represent an energy barrier within ion translocation, which is significantly reduced in the N297D variant.^34,55^ In a similar fashion, we propose that this energy barrier remains, in the best case, unaffected or increases in the N225A variant. Yet, the putative main interaction partner for the calcium ion (E54) is preserved, and the required flipping motion is still possible, resulting in the observed calcium-related kinetic effects. This once again highlights the critical role of residue E54 in VirChR1 and its calcium interaction.

*Gt*ACR1 variants E68Q and N239Q remained selective for anions and showed similar photocurrents.^14^ Especially in the case of the investigated N225A variant, this appears unlikely according to our time-resolved spectroscopic data due to the significant deceleration of all photocycle steps. Although this has to be interpreted with care. Therefore, functional studies of the variants in a calcium-dependent manner would be highly beneficial for a deeper understanding of the calcium interaction of VirChR1.

### Calcium Dependence of the Retinal Reisomerization Dynamics

**Figure 7:**
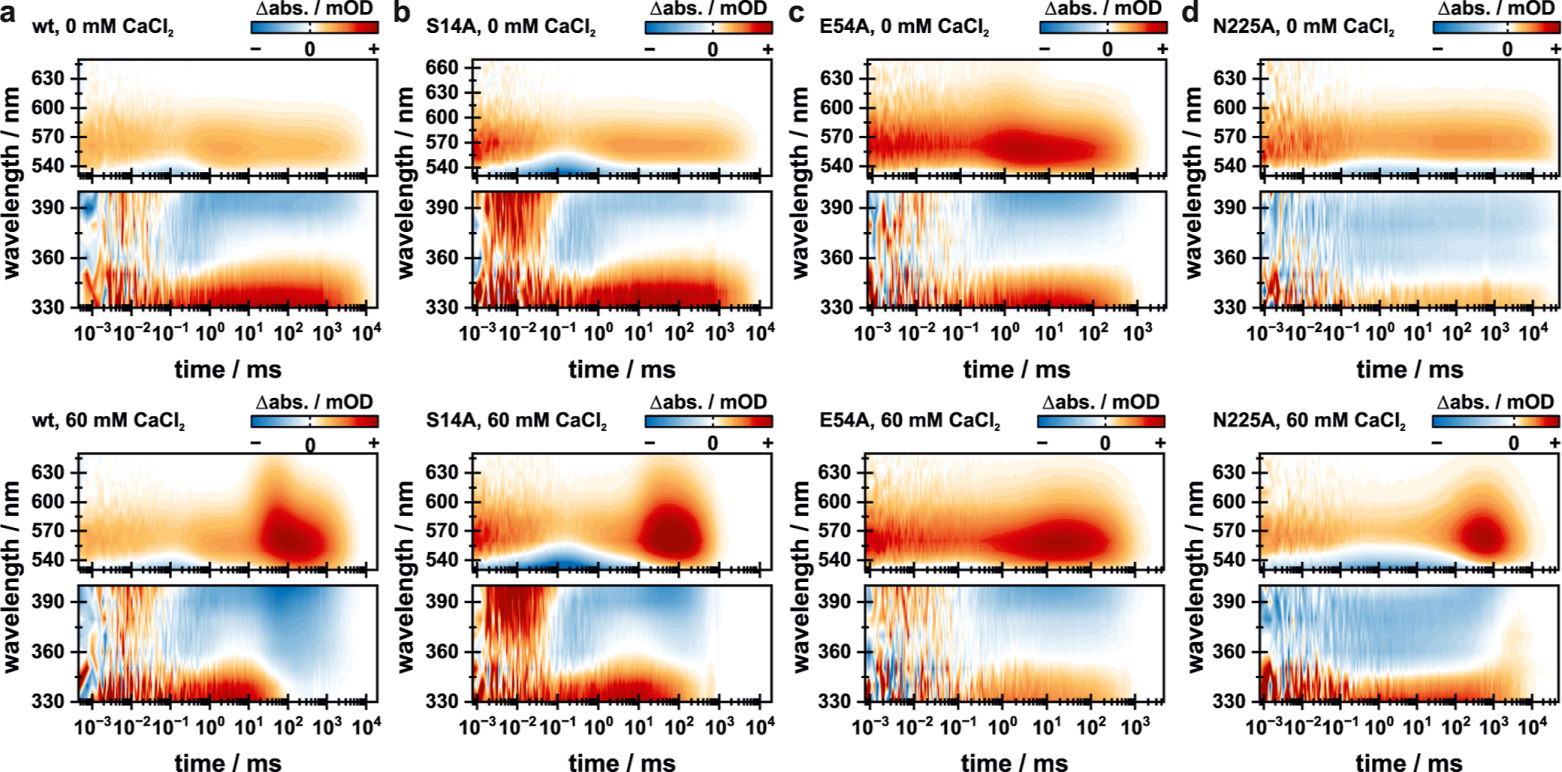
Expanded view of the photocycle dynamics in the range of 330 to 400 nm (SBS) and in the range of 530 to 650 nm (red-shifted intermediates) for the (a) wt (data taken from Lamm et al., *J. Phys. Chem. Lett.*, **2024**, *15*, 5510–5516^26^), (b) S14A variant, (c) E54A variant, and (d) N225A variant. Data in the absence of CaCl_2_ are shown on top, while the data in the presence of 60 mM CaCl_2_ are shown at the bottom. All panels in the range of 530 to 650 nm are shown in the same color code as in Figures 3 and 5, or in the respective original publication. For the 330 to 400 nm range, the color code was set to one centered around 0 mOD, spanning from −7.5 mOD to +7.5 mOD for good visibility of the SBS signature.

An important point, yet not discussed in detail, is the calcium-dependent behavior of the SBS kinetics in all investigated variants since the SBS represents a reliable spectroscopic tool for elucidating the retinal configuration. In our previous study, a calcium-dependent acceleration of the SBS decay was observed, temporally in line with the rise of the first calcium-dependent photocycle intermediate P_6_^Ca^ (Figure 7a). It was concluded that transient calcium binding, likely in close proximity to the CG, results in spatial occupation and triggers reisomerization of the retinal chromophore toward the all-trans configuration to reduce sterical hindrance and account for the changed electrostatic environment.^26^ This hypothesis is further supported by the data presented in this study. For the S14A variant, a wt-like calcium dependence was observed. Solely, the strength of the effects was reduced, which can be correlated with a reduced calcium affinity. In line with that, the SBS showed the same calcium-dependent acceleration of its decay temporally synchronized with the transition of the calcium-related photocycle intermediate P_6_^Ca^ to P_7_ (Figures 7a,b and S7). The E54A variant was identified to be almost calcium-independent, which is also reflected in the observed SBS kinetics (Figure 7c). For all investigated calcium concentrations, the SBS decay is temporally synchronized with the decay of the final P_6_ intermediate. Therefore, calcium does not trigger an earlier reisomerization of the retinal chromophore. Accordingly, reduced sterical stress and less impacted electrostatics are assumed, and the retinal chromophore stays in its 13-cis configuration. In accordance with its overall photocycle kinetics, the N225A variant again takes a special role within the investigated variants when analyzing the SBS kinetics (Figure 7d). In the absence of calcium, the usual behavior is observed. The SBS is populated throughout the photocycle and decays during the repopulation of the parent state. Under blocked channel conditions, the photocycle intermediates show a clear effect resulting in altered photocycle kinetics. This is the case for the SBS as well, while being different compared to those of the other samples. The decay of the SBS is temporally still in line with the end of the photocycle, while the P_6_ to P_7_ transition is accompanied by a spectral broadening of the SBS, resulting in a signature spanning from 330 to 380 nm. This is indicated by the negative lifetime distribution in the range of 340–400 nm centered at ≈1.4 s (Figure S6). Afterward, the SBS, now spanning from 330 to 380 nm, decays together with the final photocycle intermediate P_7_ at ≈5.2 s. Originally, the SBS was reported to be insensitive to changes in the retinal environment.^43^ More recent studies revealed that the SBS might be sensitive toward transient ion binding in the direct vicinity of the retinal chromophore.^44^ As shown by the photocycle dynamics, in both the absence and presence of calcium, the introduction of the N225A point mutation leads to strong kinetic effects. The calcium-related effects on the late red-shifted intermediates make transient calcium binding still likely, while the alteration of the CG causes changes to the observed calcium sensitivity. Due to the changed spectral and kinetic behavior of the intermediates P_5_ and P_6_, as well as the SBS, an alternative calcium-binding site likely causes an altered influence on the retinal chromophore. Yet, the strong calcium-dependent effects on the photocycle kinetics imply that even the altered interaction is located in rather close proximity to the retinal chromophore, although a calcium-dependent retinal reisomerization is not triggered.

## Conclusions

Time-resolved UV/vis spectroscopic experiments on the late nanosecond to second time scale of VirChR1 variants S14A, E54A, and N225A reveal their key role in both general photocycle characteristics and calcium dependence of VirChR1. Changes in the amplitudes of intermediates P_2_, P_3_, and P_4_, accompanied by unaffected lifetimes for associated intermediate state transitions, further support the existence of equilibria in between the mentioned intermediates during the photocycle, as observed for C1C2.^45^ The SEN-triad was identified to strongly affect RSB deprotonation, while the impact is stronger for SEN-triad members S14 and N225 compared to E54. Considering the findings of VanGordon et al.,^52^ we concluded that hydrogen bonding network alterations induce p*K*_a_ value changes, thus facilitating RSB deprotonation. Although our data cannot identify whether transient calcium binding occurs within the SEN-triad, the central role for the calcium sensitivity was proven. A minor role was assigned to residue S14. In contrast, E54 was identified as a key residue for calcium sensitivity. This is in line with E54 being the only SEN-triad member with a carboxylate group, which plays a main role in ion translocation in C1C2.^55^ The role of residue N225 within the SEN-triad varied significantly from those of the other members. Lack of the P_3_ intermediate suggests either direct or indirect involvement of N225 in structural changes associated with this transition. The functionality of this variant is highly in doubt due to the alteration of the photocycle kinetics, while a direct comparison between photocycle dynamics and protein function has to be carefully checked, as in *Cr*ChR2 R120H.^58^ Yet, the calcium dependence of N225A is significantly different from the previously observed ones. The spectral positions of calcium-binding-related intermediates differ, as indicated by an altered modulation of GSB intensity. In addition, the response of SBS to the presence of calcium is changed completely. Calcium usually forces an earlier SBS decay, temporally synchronized with the calcium-related effects on the photocycle kinetics. Accordingly, it is concluded that transient calcium binding promotes retinal reisomerization toward the all-trans configuration.^26^ For the N225A variant, the SBS spectrally broadens during the P_6_ to P_7_ transition, indicative of either a potential additional calcium-binding site in VirChR1 or an altered release mechanism of the calcium ion since a calcium-bound species was not accumulated during the course of the measurement in either case. The calcium ion has to influence the spectroscopic properties of 13-cis retinal in a different way. Most likely, it passes the chromophore in closer proximity. Mous et al.^59^ showed that ions located in close proximity to the retinal chromophore interact with the respective π-electron system. Such interactions have already been linked to changes in SBS behavior.^44^

The insights into the calcium sensitivity of VirChR1 presented in this study further confirm the key role of SEN-triads and the CG on the ion dependence of cation channelrhodopsins. The observed trends provide the first starting points for engineering VirChR1 calcium selectivity. The variants investigated in this study caused a decrease in the calcium sensitivity. For application in optogenetics, an increase of calcium sensitivity toward the low physiological calcium concentration might be desirable. Our data provide the first starting points for this approach since we identified residues that tune the calcium affinity, and suitable mutations should increase calcium affinity. According to published work on *Cr*ChR1 and C1C2,^55^ a N225D variant would be a prime candidate for this approach. Yet, this whole process would highly benefit from additional functional and structural data, which are up to future studies of VirChR1.

## Supporting Information Available

Predicted local distance difference test score for the calculated models of VirChR1 opsin, comparison of noncorrected and artifact-corrected data, kinetic analysis of measurements at 0 mM and 60 mM CaCl_2_, additional transients for the N225A variant in the presence of 60 mM CaCl_2_, closer view on the SBS dynamics of the wt at CaCl_2_ concentration 1 mM and 4 mM, and comparison of LDMs highlighting the pattern describing intermediates P_3_ and P_4_ (PDF)
