# Supplementary material for: Combined Mutational and Spectroscopic Study on the Calcium-Related Kinetic Effects on the VirChR1 Photocycle
Source: J Phys Chem B. 2025 Mar 10;129(11):2946–57. doi: 10.1021/acs.jpcb.4c08416 (PMC11931529; doi:10.1021/acs.jpcb.4c08416)
Supplement: Supplementary file 1 — jp4c08416_si_001.pdf [file jp4c08416_si_001.pdf]

Supporting Information
Combined Mutational and Spectroscopic Study on the Calcium Related Kinetic Effects on the VirChR1 Photocycle

Gerrit H. U. Lamm,[†] Dmitrii Zabelskii,[‡] Taras Balandin,[¶,§] Valentin Gordeliy,[¶,§,‖] and Josef Wachtveitl[*,†]

†*Institute of Physical and Theoretical Chemistry, Goethe University Frankfurt, 60438, Frankfurt am Main Germany*

‡*European XFEL, 22869, Schenefeld, Germany*

¶*Institute of Biological Information Processing (IBI-7: Structural Biochemistry), Forschungszentrum Jülich, 52428 Jülich, Germany*

§*JuStruct: Jülich Center for Structural Biology, Forschungszentrum Jülich, 52428 Jülich, Germany*

‖*Univ. Grenoble Alpes, CEA, CNRS, Institute de Biologie Structurale (IBS), 38000, Grenoble, France*

E-mail: wveitl@theochem.uni-frankfurt.de

Supplementary Data

Structure Prediction

Table S1: Parameters used for the structure prediction.

Parameter	Value	Parameter	Value
msa_method	mmseqs2	use_ptm	True
homooligomer	2	rank_by	pLDDT
pair_mode	unpaired	num_models	3
cov	0	num_samples	1
qid	0	num_ensemble	1
max_msa	512:1024	max_recycles	3
subsample_msa	True	is_training	False
num_relax	1	use_templates	False
use_turbo	True		

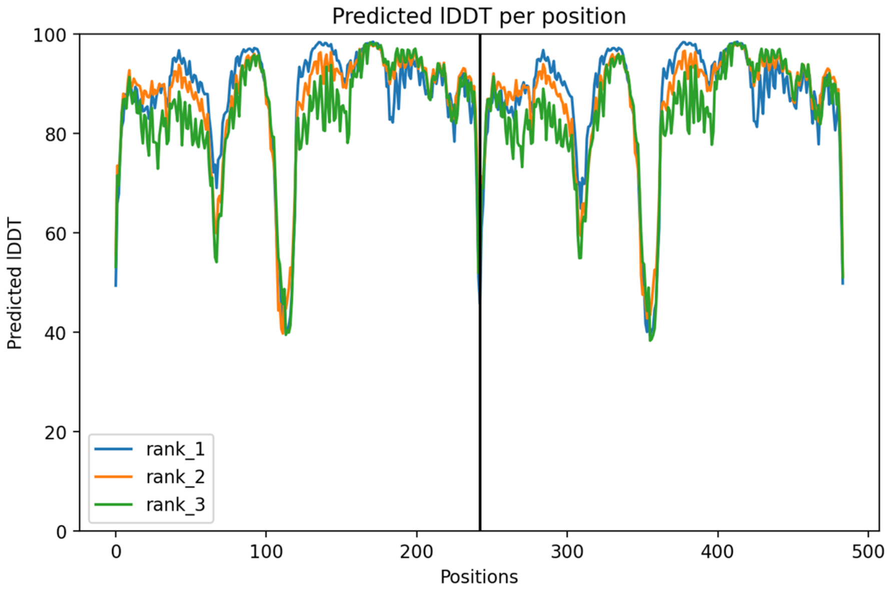

Figure S1: Predicted local distance difference test score for the calculated models of VirChR1 opsin.

Comparison of artifact corrected and non-corrected flash photolysis data

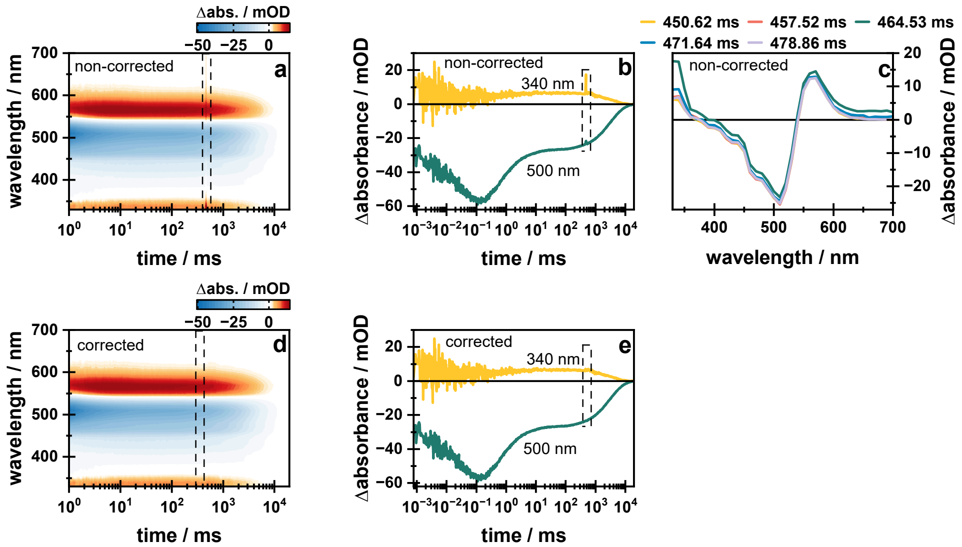

Figure S2: **Exemplary comparison of non-corrected and artifact corrected for VirChR1 S14A under permeable channel conditions.**

The S14A variant

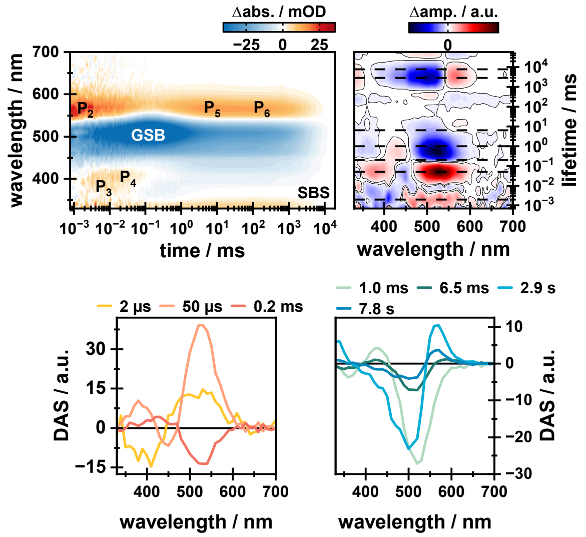

Figure S3: ns to s dynamics of VirChR1 S14A at pH 8.0 and 0 mM $CaCl_2$ (top left) together with the corresponding LDM (top right) and DAS (bottom) to retrieve the kinetic information of the measurement.

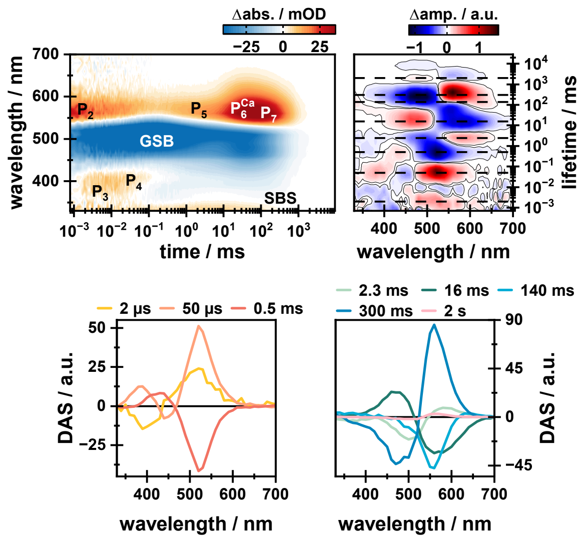

Figure S4: ns to s dynamics of VirChR1 S14A at pH 8.0 and 60 mM $CaCl_2$ (top left) together with the corresponding LDM (top right) and DAS (bottom) to retrieve the kinetic information of the measurement.

The E54A variant

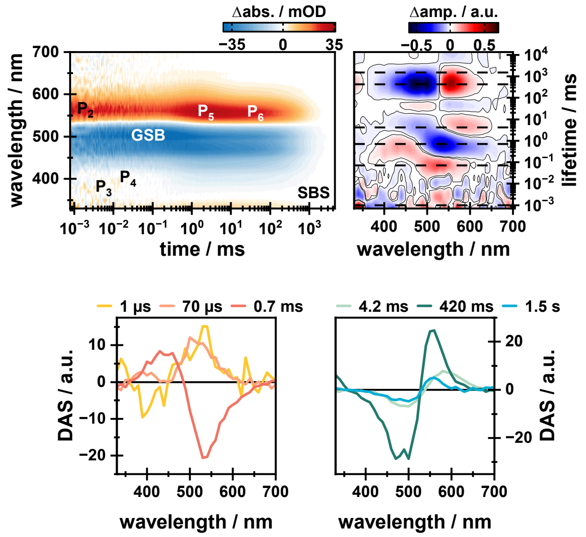

Figure S5: ns to s dynamics of VirChR1 E54A at pH 8.0 and 0 mM $CaCl_2$ (top left) together with the corresponding LDM (top right) and DAS (bottom) to retrieve the kinetic information of the measurement.

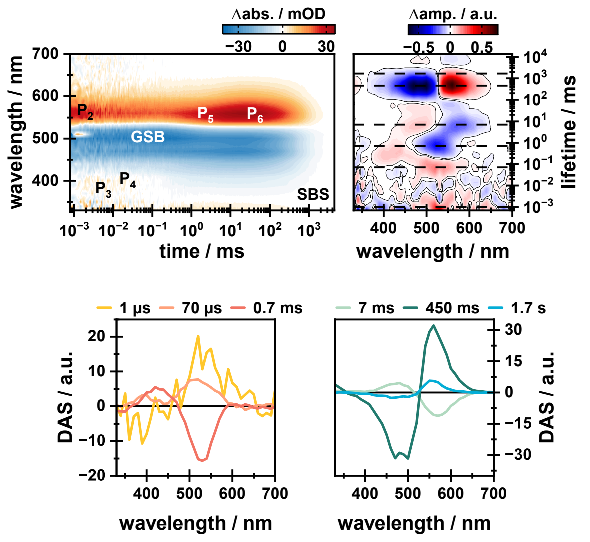

Figure S6: **ns to s dynamics of VirChR1 E54A at pH 8.0 and 60 mM $CaCl_2$ (top left) together with the corresponding LDM (top right) and DAS (bottom) to retrieve the kinetic information of the measurement.**

The N225A variant

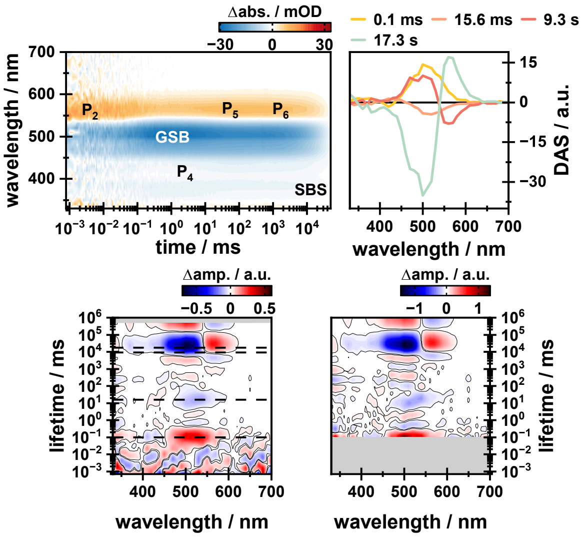

Figure S7: ns to s dynamics of VirChR1 N225A at pH 8.0 and 0 mM $CaCl_2$ (top left) together with the corresponding LDM (top right) and DAS (bottom) to retrieve the kinetic information of the measurement.

For this measurement the LDM has been splitted, due to the temporal range of the measurement (7.84 ns to 49.5 s). Additionally, signal amplitudes are almost constant for a certain time interval, resulting in the observed oscillation features. The LDM shown in the left faced issues in describing the signal behavior at the very end, due to the limited amount of lifetimes set in OPTIMUS software. Therefore, a second LDM focusing on this part of the measurement was calculated. Both LDMs are shown in the same temporal range, and missing parts are displayed in gray. Both LDMs being complementary in their retrieved kinetic features supports the used approach, as well as data analysis via LDA in general.

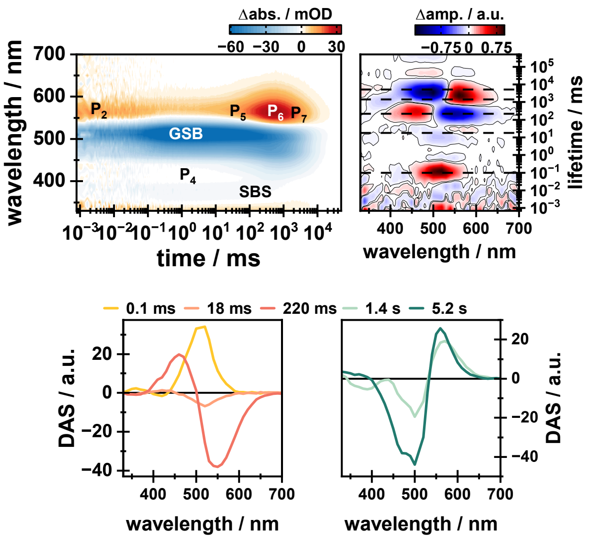

Figure S8: ns to s dynamics of VirChR1 N225A at pH 8.0 and 60 mM $CaCl_2$ (top left) together with the corresponding LDM (bottom) and DAS (top right) to retrieve the kinetic information of the measurement.

Lifetime Comparison

Table S2: Lifetimes determined for variants S14A, E54A, and N225A under permeable channel conditions.

Sample	τ_1	τ_2	τ_3	τ_4	τ_5	τ_6	τ_7
S14A	2 µs	50 µs	0.2 ms	1.0 ms	6.5 ms	2.9 s	7.8 s
E54A	1 µs	70 µs	0.7 ms	—	4.2 ms	420 ms	1.5 s
N225A	0.1 ms	—	—	—	15.6 ms	9.3 s	17.3 s

Table S3: Lifetimes determined for variants S14A, E54A, and N225A under blocked channel conditions.

Sample	τ_1	τ_2	τ_3	τ_4	τ_5	τ_6	τ_7	τ_8	τ_9
S14A	2 µs	50 µs	0.5 ms	—	2.3 ms	16 ms	140 ms	300 ms	2.0 s
E54A	1 µs	70 µs	0.7 ms	—	7 ms	450 ms	1.7 s		
N225A	0.1 ms	—	—	18 ms	220 ms	—	—	1.4 s	5.2 s

LDM comparison

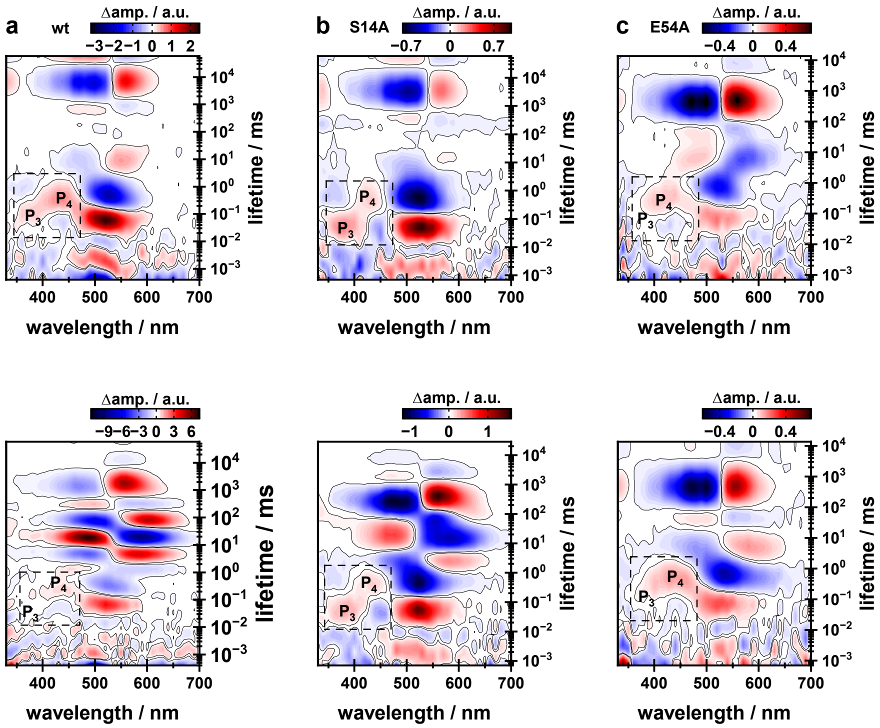

Figure S9: Comparison of LDMs for **a)** the wt, and variants **b)** S14A, and **c)** E54A. Permeable channel conditions are shown in the top row, while blocked channel conditions are shown in the bottom row. The pattern resolving the kinetics of intermediates P_3 and P_4 is highlighted with a dashed box. The large amplitudes of lifetime distributions related to the strong calcium related signals, may make the pattern hard to observe due to the color coding.

WT measurements for comparison

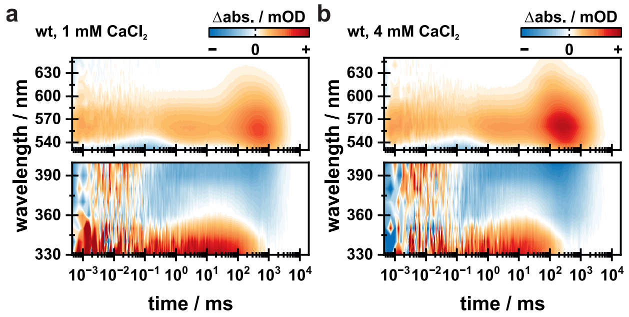

Figure S10: Expanded view on the photocycle dynamics in the range of 330 nm to 400 nm (SBS) and in the range of 530 nm to 650 nm (red-shifted intermediates) for **a)** the wt at 1 mM $CaCl_2$ and **b)** 4 mM $CaCl_2$ (data taken from Lamm et al., *J. Phys. Chem. Lett.*, **2024**, *15*, 5510-5516[1]). All panels in the range of 530 nm to 650 nm are shown in the same color code as in the respective original publication. For the 330 nm to 400 nm range, the color code was set to one centered around 0 mOD, spanning from -7.5 mOD to +7.5 mOD for good visibility of the SBS signature.

References

(1) Lamm, G. H. U.; Zabelskii, D.; Balandin, T.; Gordeliy, V.; Wachtveitl, J. Calcium-Sensitive Microbial Rhodopsin VirChR1: A Femtosecond to Second Photocycle Study. *J. Phys. Chem. Lett.* **2024**, *15*, 5510–5516.
